# In-line fluorescence characterization of sodium fluorescein and application as tracer in residence time distribution analysis at elevated temperatures

**DOI:** 10.1016/j.mex.2026.103925

**Published:** 2026-04-21

**Authors:** Dominik Hug, Jonas Meury, Joseph Dumpler, Corina Sägesser, Alexander Mathys

**Affiliations:** Sustainable Food Processing Laboratory, Department of Health Science and Technology, ETH Zurich 8092 Zurich, Switzerland

**Keywords:** Sodium fluorescein, Fluorescent tracer, Tracer characterization, Fluorescence spectroscopy, Continuous thermal processing, Residence time distribution

## Abstract

Residence time distribution (RTD) characterization to validate continuous thermal processing systems at elevated temperatures is an important metric and particularly relevant for ultra-high temperature (UHT) and high-temperature short-time (HTST) treatments. Sodium fluorescein (SF) was investigated as a fluorescent tracer for RTD measurements at temperatures extending beyond previously reported ranges. Using a custom-built flow-through measurement system, fluorescence intensity and emission spectra were recorded from 65 - 136 °C in a stabilized pH 7.4 phosphate buffer. Linear thermal quenching ($\alpha_{S}$ = -0.54%·K⁻¹, 95% CI: 0.554 to -0.535) and bathochromic shift (0.06 nm·K⁻¹) were quantified and no significant thermal degradation up to 145 °C was identified. Step-response experiments at 25 - 105 °C demonstrated that temperature-normalized fluorescence signals yield consistent cumulative distribution functions across the thermal range investigated, with mean residence times obtained using a semi-empirical beta-distribution model. The independently measured temperature sensitivity from RTD experiments ($\alpha_{RTD}\;$= -0.56%·K⁻¹) agreed well with spectral measurements. These results establish a quantitative framework for temperature-invariant RTD characterization in non-isothermal flow-through reactors with SF as tracer.

## Highlights

• Sodium fluorescein exhibits no significant thermal degradation up to 145 °C.
• Thermal quenching (−0.54%·K⁻¹) and bathochromic shift of sodium fluorescein were quantified from 65 to 136 °C.
• Temperature-normalized fluorescence allowed robust RTD characterization across a range of 25 to 105 °C.

## Graphical abstract

**Figure:**
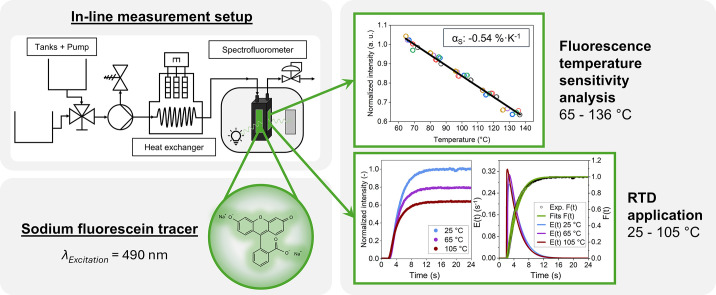

## Specifications table

**Table:** 

Subject area	Chemical Engineering
More specific subject area	Thermal Process Engineering
Name of your method	In-line fluorescence characterization of sodium fluorescein and application as tracer in residence time distribution analysis at elevated temperatures
Name and reference of original method	J.H. Ham, B. Platzer, Semi-empirical equations for residence time distributions in disperse systems - Part 1: Continuous phase, Chem. Eng. Technol. 27 (2004) 1172–1178.
Resource availability	Resource specifications: • LS50B spectrofluorometer, PerkinElmer Inc., Waltham, MA, USA • Polystyrene cuvettes, Sarstedt AG & Co. KG, Nümbrecht, Germany • MCP-Z Standard gear pump drive (ISM 405), Ismatec SA, Glattbrugg, Switzerland • Flow-through cuvette (176–766–15–40), Hellma Analytics, Müllheim, Germany • Julabo ME oil bath, JULABO GmbH, Seelbach, Germany • FL WinLab software, PerkinElmer Inc., Waltham, USA • MATLAB R2024b with Optimization Toolbox, The MathWorks Inc., Natick, USA • Spectragryph v1.2.16.1, Spectroscopy Ninja, Dummelsmoos, Germany • R v4.5.2, R Foundation for Statistical Computing, Vienna, Austria • Sodium fluorescein, CAS 518–47–8, Fisher Scientific, Loughborough, UK

## Background

Residence time distribution (RTD) characteristic is a key parameter for characterizing continuous chemical or thermal processes, its determination becomes a requirement in thermal treatments to ensure product safety and quality through determination of minimum, average, and maximum residence times. Therefore, a comprehensive analysis of the reactor's flow profile should be conducted prior to its implementation in process development [1]. Modern food and pharmaceutical processing increasingly rely on high-temperature short-time (HTST) thermal treatments or ultra-high temperature (UHT) processing to achieve microbial inactivation and produce shelf-stable products with extended microbiological stability [2]. Beyond conventional heat exchangers, emerging reactor designs exploit indirect heat transfer characteristics within compact geometries. These systems enable processing windows that minimize thermal damage while maximizing the retention of heat-sensitive nutritional and organoleptic properties and preserving microbial safety [3]. Experimental validation of reactor designs operating within these regimes necessitates RTD measurements conducted at elevated temperatures.

Sodium fluorescein (SF) has been used as a tracer across a wide range of research fields, including hydrology, biomedical assays, environmental tracing and microfluidics [[4], [5], [6]]. Due to its high quantum yield, high water solubility and non-invasive measuring capabilities [1,[7], [8], [9]], SF has been identified as a promising candidate for fluorescent tracer in RTD analysis. SF has been used as a diagnostic agent in clinical medicine, with a well-documented safety profile [10]. While SF behaviour is well-documented near room temperature [11,12], data on its fluorescence characteristics at elevated temperatures (>80 °C) remain sparse. Consequently, most available literature on fluorescence of tracer substances focuses on its characteristics at lower temperatures (<80 °C) or on its thermal stability rather than on its fluorescence behavior during detection at elevated temperature (>80 °C). Wang’ombe et al [13] used SF as a geothermal tracer where the substance temporarily underwent 250 °C and measurement was conducted once the carrier fluid was cooled down to room temperature again. Guan and Su [14] studied the fluorescence emission of SF bound to starch up to 60 °C describing a decrease of SF fluorescence intensity and a red shift towards higher temperatures. Sjöback et al [15] investigated the absorbance of SF to a temperature of 80 °C and found a shift of the absorption peak of 0.07 nm K^-1^. SF has also been employed in laser-induced fluorescence (LIF) studies [11,16]. Recently, fluorescence characterization of SF in the temperature range of 10 - 60 °C for usage in dual-emission laser-laser induced fluorescence (DELIF) systems were investigated by Toriyama et al [17]. Modern optical systems enable high-frequency data acquisition with temporal resolutions down to 2 ⋅ 10⁻⁶ s [18] and beyond, making fluorescence tracers particularly well-suited for rapid, online detection in millisecond residence time measurements.

This study adopts established RTD modeling frameworks [1,19] to enable temperature-invariant characterization of flow-through microreactors operating under non-isothermal conditions. We present an experimental investigation of SF fluorescence intensity and spectral peak shift in a pH-stabilized aqueous solution using a custom-built high-temperature measurement setup, thereby extending the characterization range beyond previous literature reports. Building on this, we propose a validation approach to account for thermal quenching effects during RTD tracer measurements in flow-through reactor systems with temperature gradients.

## Method details

### Fluorescent dye properties and preparation

Although several fluorescent dyes are suitable for RTD characterization, their applicability in sensitive industrial contexts such as food processing and pharmaceutical manufacturing is constrained by safety requirements. Beyond toxicological considerations, the selected tracer must exhibit favorable optical detection properties under process-relevant conditions. Sutton et al [12] analyzed different fluorophores for their application of Laser-Induced Fluorescence (LIF) for thermometry in aqueous flows and highlighted the constraints by five fundamental requirements regarding the tracer and optical setup. First, the fluorescent tracer must possess high water solubility. In the case of SF, a quenching effect occurs prior to the saturation concentration being reached. Second, it must demonstrate a quantifiable temperature sensitivity across the thermal range of interest. However, if sensitivity decreases with increasing temperature, the dependence should not be too high, since precise detection becomes more difficult with lower sensitivity. Third, the dye requires sufficient photostability to withstand the excitation intensities needed for detection without significant photobleaching. Choosing a favorable dye absorption wavelength and suitable concentration, governed by the short exposure time reduces required excitation source power and thus precludes photobleaching. Fourth, there must be adequate spectral separation between the absorption and emission bands, i.e., the Stokes shift must be large enough, to facilitate signal discrimination. Finally, re-absorption of the emitted fluorescence by the solution itself (inner filter effect) must be minimized through careful path length and concentration management.

While factors such as solubility, photostability, spectral separation, and re-absorption are largely determined by the specific experimental setup or are established for dyes like SF [5,12,17], the requirement for temperature sensitivity necessitates further examination for high-temperature short-time conditions such as UHT and indirect heat transfer systems.

The fluorescence intensity of SF depends on the pH of the solution [[20], [21], [22]]. Water undergoes pH changes when heated, primarily due to the temperature dependence of the ion product (K_w_), which increases with temperature, causing both pH and pOH of pure water to decrease while the solution remains neutral [23,24]. To prevent temperature-induced pH variations from confounding the temperature-dependent fluorescence measurements, pH stabilization using a buffer is mandatory. The PBS buffer solution showed minimal pH change of −0.03 ± 0.03 in the temperature range of 20 - 130 °C [25], making it ideal for high temperature short-time treatment measurements. Therefore, all experiments were conducted with 0.05 M PBS adjusted to pH 7.4 to maintain the pH across the experimental temperature range. A 0.2 M phosphate-buffered saline (PBS) stock solution was prepared by dissolving 92.07 g Na₂HPO₄ and 18.24 g KH₂PO₄ in 4.00 kg of ultrapure water under stirring at room temperature. This stock solution was subsequently diluted with ultrapure water to prepare 0.05 M PBS working buffers at final volumes of 5 L or 15 L, each prepared from the appropriate portion of the 0.2 M stock solution. The pH was adjusted to 7.40 using 1 M HCl. A 1 g·L^-1^ SF (CAS 518–47–8; Fisher Scientific, Loughborough, UK) stock solution was prepared by dissolving 100 mg of SF in 100 mL of 0.05 M PBS and storing the solution in a light-protected container. Final SF working solutions were prepared by diluting the SF stock solution with 0.05 M PBS (pH 7.40) to obtain concentrations of 0.5 mg·L^-1^ SF. All solutions were stored in sealed, light-resistant containers at room temperature and used on the same day.

### Definition of operation windows with concentration-series tests

A preliminary concentration-series experiment was conducted to characterize concentration-dependent red shifts and self-quenching of SF. A 6140 mg·L⁻¹ stock solution was prepared in 0.05 M PBS (pH 7.40) and subsequently diluted in a 1:3 serial dilution over 11 steps, yielding a final concentration of 0.0234 mg·L⁻¹ SF. Fluorescence measurements were performed using a microplate reader (Spark, Tecan Group Ltd., Männedorf, Switzerland) in a 96-well plate with excitation wavelength of 490 nm, excitation and emission bandwidths of 5 nm, and emission spectra recorded from 500 to 650 nm. Each concentration was dispensed into four wells, resulting in four technical replicates per concentration. A blank consisting of 0.05 M PBS was measured and subtracted from each spectrum. The same concentration series was additionally measured using a LS50B spectrofluorometer (PerkinElmer Inc., Waltham, MA, USA) equipped with polystyrene cuvettes (Sarstedt AG & Co. KG, Nümbrecht, Germany) to determine concentration-dependent spectral red shifts. On the same device, a second concentration series was prepared to determine the linear relationship between SF concentration and fluorescence emission under the measurement and continuous system conditions similar to the RTD setup. A 2000 mg·L⁻¹ SF stock solution was prepared in 0.05 M PBS solution (pH 7.4). From this stock, a 2 mg·L⁻¹ working solution was prepared and subjected to a four-step twofold serial dilution, resulting in a final concentration of 0.125 mg·L⁻¹. The measurements were executed with an excitation wavelength of 490 nm, an excitation and emission slit widths of 4 nm, and a scan speed of 300 nm·min⁻¹.

### Experimental setup of continuous fluorescence measurement

**Fig. 1:**
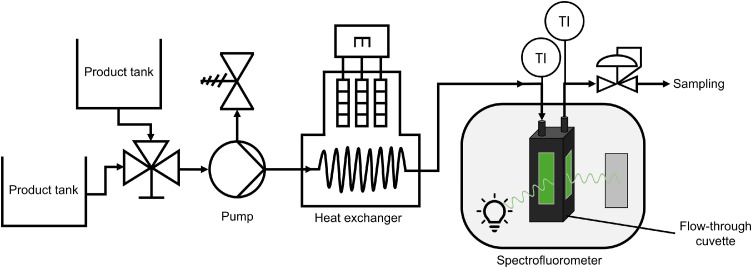
Schematic diagram of the experimental setup for continuous, in-line fluorescence measurement. The system consists of a dual-tank feed connected via a switching valve to a pump, which circulates the fluid through a heat exchanger and into a spectrofluorometer housing a flow-through cuvette for fluorescence signal detection.

A custom-built optical measurement setup (Fig. 1) was employed to record the fluorescence intensity of SF using a flow-through cuvette, allowing continuous measurements under elevated-temperature conditions.

**Fig. 2:**
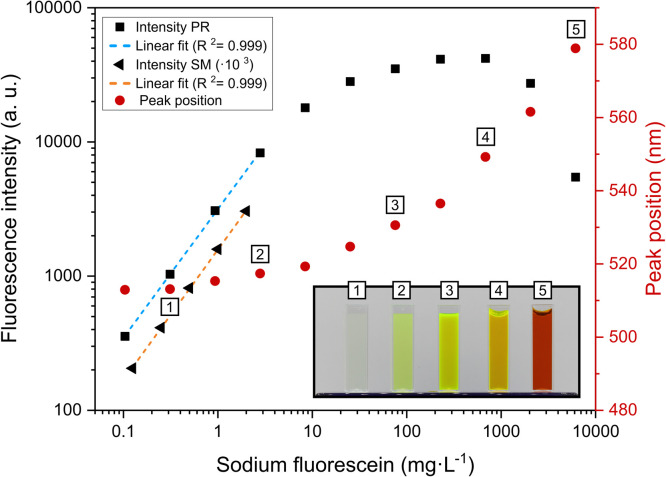
Fluorescence intensity measured with plate reader (PR) and spectrofluorometer (SM) as a function of sodium fluorescein concentration. The dashed lines represent linear fits at lower concentrations (R^2^ = 0.999). The inset displays photographs of the SF solutions corresponding to the data points labeled 1 - 5 (0.31, 2.8, 76, 682, and 6140 mg·L⁻¹, respectively).

Fluorescence intensity varies linearly with SF concentration up to 8.4 mg·L^-1^. Above this concentration the signal response began to taper off, forming a plateau that peaked at 682 mg·L^-1^ attributable to measurement sensor saturation and onset of self-quenching (Fig. 2). Above 682 mg·L⁻¹, the decrease in fluorescence intensity is then solely attributed to the self-quenching of SF molecules [[20], [26]]. Fluorescence intensity characterization performed by Walker [27] and Käss [22] showed linearity with concentration over a range of nearly 5 decades for low concentration, followed by formation of a plateau and a decrease in intensity at very high concentrations. Maintaining experimental conditions within this linear range is crucial for accurate RTD analysis. The plateau and quenching regions observed at higher concentrations are unsuitable for quantitative analysis, as they would result in a considerable underestimation of the true tracer concentration. To ensure accuracy in the subsequent temperature-dependency experiments, a tracer concentration within the identified linear regime was selected (0.5 mg·L^-1^). Liu et al [20] stated a shift to longer wavelengths of the SF peak intensity, with increasing concentration in a range of 0.38 to 3762 mg·L^-1^ and observed a shift from 510 nm to 550 nm with an excitation at 470 nm. Similarly, we report a peak shift of SF when increasing the concentration in the solution. The emission peak for the lowest concentration (0.1 mg·L^-1^) was at 513 nm, while the peak for the highest concentration (6140 mg·L^-1^) is at 579 nm (Fig. 2). The inset in Fig. 2 qualitatively illustrates this red shift, demonstrating a distinct transition in solution appearance from green at low concentrations to red at high concentrations. The spectral characteristics have two primary implications for RTD assessment. In configurations where the narrow Stokes shift (∼25 nm) presents challenges, such as an overlapping signal, increasing the SF concentration may partially mitigate these effects. However, the concentration-dependent red shift necessitates appropriate selection of the detection wavelength and bandpass filters to prevent signal attenuation resulting from the emission peak drifting outside the filter transmission window.

The setup included two reservoirs (1 L SF solution; 2 L deionized water) connected via stainless-steel tubing (ID: 1.76 mm, OD: 3.18 mm) to a three-way valve. The respective selected fluid stream was then conveyed by a MCP-Z Standard gear pump drive (ISM 405) (Ismatec SA, Glattbrugg, Switzerland) through coil windings (16 turns, Ø 70 mm) submerged in a temperature-controlled oil bath (Julabo ME, Julabo GmbH, Seelbach, Germany) for heating. Downstream of the oil bath, the pipes were thermally insulated with glass wool and routed to a custom double-T connector that directed the flow to the flow-through cuvette. The flow-through cuvette (176–766–15–40) (Hellma Analytics, Müllheim, Germany) was positioned inside the cuvette holder of the LS-50B spectrofluorometer for fluorescence detection. Measurements were always performed at 10 mm path length. The fluid temperature was monitored at the cuvette inlet ($T_{1}$) and outlet ($T_{2}$) using two K-type thermocouples with 0.5 mm diameter (IEC Class 1) (Thermocoax, Athis-de-l'Orne, France). Owing to the symmetrical configuration of the thermocouples, the spectral data were paired with the mean fluid temperature, assumed to be the average of $T_{1}$ and $T_{2}$. Downstream of the cuvette, the fluid flow was guided through a cooling coil (13 turns, Ø 55 mm) submerged in an ice water bath, and subsequently a backpressure valve set to 8 bar.

### In-line fluorescence spectra data acquisition procedure

The measurement setup was preheated by circulating deionized (DI) water at the target temperature. Once a steady-state temperature was reached, the flow was switched from DI water to the SF solution. Measurements were taken once the spectrofluorometer signal stabilized, indicating steady-state fluorescence intensity.

Spectral data were acquired using FL WinLab software (PerkinElmer Inc., Waltham, USA) and further processed in Spectragryph software (version 1.2.16.1, Spectroscopy Ninja, Dummelsmoos, Germany). Fluorescence spectra were recorded for a temperature range of 65 to 136 °C using the LS50B spectrofluorometer under constant excitation (excitation wavelength 490 nm, emission range 505 - 550 nm, scanning speed 500 nm·min⁻¹, and excitation and emission slit widths of 5 nm). Four individual measurements were captured for each temperature, while each measurement consisted of an approximately 10 s acquisition time. An excitation wavelength of 490 nm was selected, as it closely matches the absorption maximum of SF at 488 nm while coinciding with the commercially available emission wavelength of fiber-coupled LED sources predestined for in-line RTD measurements. This approach eliminated the requirement for laser-based excitation, thereby avoiding additional safety protocols and shielding requirements associated with laser systems, yet providing comparable results. The pump speed was held constant at a constant mass flow of 108 g·min⁻¹ which was determined by collecting the effluent for 30 s. Samples were immediately stored on ice for subsequent heat-degradation analysis.

Following each measurement, the system was switched back to DI water, which continued circulating while the oil bath was heated to the next target temperature. Once thermal steady state was re-established, the flow was again switched to the SF solution and the measurement repeated. For each experimental series, runs were carried out in the temperature ramp-up and ramp-down procedure. This entire sequence of measuring procedure was performed in triplicate on three separate days.

### Heat degradation analysis

After every temperature step of each run, samples were collected for heat-degradation analysis at the outlet. For each sample, four wells of a 96-well plate were filled with 200 µL of solution. An unheated sample of the SF solution was also collected and served as control. The fluorescence intensity was measured using the microplate reader. Measurements were performed at an excitation wavelength of 490 nm (5 nm bandwidth) and an emission wavelength of 516 nm (10 nm bandwidth).

### Mathematical framework for fluorescence intensity dependence

To ensure robust quantification of the temperature dependence of SF fluorescence, the emission spectra were integrated over a range of 510 - 530 nm. Statistical analyses were conducted in R software (version 4.5.2, R Foundation for Statistical Computing, Vienna, Austria). A non-linear mixed-effects model was applied to account for run-to-run variations in absolute signal intensity while extracting a single global temperature sensitivity coefficient ($\alpha_{S}$). The model is defined as:

(1) $$\begin{array}{c} I_{i}\left(T\right)=A_{i}\cdot \left[1+\alpha_{S}\cdot \left(T\;-\;T_{\mathrm{ref}}\right)\right] \end{array}$$

where $I_{i}\left(T\right)$ is the integrated intensity for experimental run i, $A_{i}$ is the run-specific scaling factor representing the baseline intensity at the reference temperature, and $T_{ref}$ is the reference temperature set to 70 °C. The model parameters were estimated using the Levenberg-Marquardt algorithm to account for minor experimental variations in tracer concentration or optical alignment.

### Residence time distribution data acquisition

RTD data was acquired similarly as described previously with the LS50B spectrofluorometer, using FL WinLab software in TimeDrive setting. The excitation wavelength was set to 490 nm (slit width of 5 nm) and emission wavelength to 517 nm with slit width of 10 nm. As data interval, corresponding to the temporal resolution, 0.02 s was chosen. The pump speed was set to a mass flow of 148 g·min^-1^. All runs were performed in triplicate.

### Mathematical framework for residence time distribution analysis

The experimental setup was designed to measure RTD directly at elevated process temperatures as proof-of-concept. The fluid was heated to target temperature, and the fluorescence signal was recorded in-situ in the measurement cell. This configuration allows for the characterization of the flow path from the injection valve to the detection point. The data processing workflow consisted of three stages: (1) temperature-invariant normalization, (2) semi-empirical parameter estimation, and (3) calculation of hydrodynamic moments.

Raw fluorescence intensity signals I(t,T) are subject to thermal quenching, resulting in reduced signal amplitudes at higher temperatures. To directly compare flow profiles across different thermal regimes (25, 65, 105 °C), the transient signals were normalized against their temperature-specific boundary conditions. For a step-input experiment switching from carrier fluid to tracer-containing solution, the cumulative RTD F(t) represents the dimensionless concentration ratio $C\left(t\right)/C_{0}$. $F_{exp}\left(t\right)$ was derived from:

(2) $$F_{exp}\left(t\right)=\frac{I\left(t,T\right)-I_{base}\left(T\right)}{I_{max}\left(T\right)-I_{base}\left(T\right)}$$

where $I_{base}\left(T\right)$ and $I_{max}\left(T\right)\;$ correspond to the steady-state fluorescence intensities of the blank carrier fluid and the tracer saturation plateau at temperature T, respectively. This yields a normalized concentration distribution $F_{exp}\left(t\right)\in \left[0,1\right]$ that represents hydrodynamic transport properties.

The independent characterization of $\alpha_{S}\;$ enables a critical mass balance verification during RTD experiments. The normalization in Eq. (2) implicitly assumes that the plateau signal $I_{max}\left(T\right)\;$ at the detector corresponds to the full injected tracer concentration. A known $\alpha_{S}\;$ allows the prediction of the expected $I_{max}\left(T\right)\;$ at any process temperature. Deviations between the predicted and measured plateau signal are therefore direct evidence of non-conservative tracer behavior, including irreversible thermal degradation, adsorption to reactor walls, or concentration losses along the flow path, deviations that are otherwise entirely masked by normalization. This constitutes an independent validation layer.

To extract robust hydrodynamic parameters without the noise amplification associated with numerical differentiation of step data E(t)=dF/dt, the experimental $F_{exp}\left(t\right)$ curves were fitted to the semi-empirical model originally proposed by Ham and Platzer [19] and subsequently applied to microchannel systems by Adeosun and Lawal [1]. This model describes the residence time density E(t) using a beta-distribution-type function, which is particularly well-suited for capturing asymmetric flow profiles typical of laminar flow systems. The beta function B(H,M) is dependent on the parameters *M* and *H*, which are shape factors governing the skewness and kurtosis of the RTD and *u* is the integration variable. The modeled cumulative distribution $F_{model}\left(t\right)$ can thus be written as:

(3) $$F_{model}\left(\theta \right)=I_{\theta }\left(H,M\right)=\frac{1}{B\left(H,M\right)}\int_{0}^{\theta }u^{H-1}{\left(1-u\right)}^{M-1}du$$

where θ is the normalized time variable defined on the residence time domain [$t_{min},t_{max}]$ as:

(4) $$\theta \left(t\right)=\frac{t-t_{min}}{t_{max}-t_{min}}$$

with $t_{min}$ representing the minimum residence time (breakthrough time), corresponding to the first detectable tracer appearance, and $t_{max}$ denoting the maximum residence time, defined as the time point where the cumulative distribution reaches saturation. The model parameters were estimated by fitting Eq. (3) to the experimental $F_{exp}\left(t\right)$ data using a non-linear least-squares algorithm (lsqcurvefit) implemented in MATLAB R2024b with Optimization Toolbox (The MathWorks Inc., Natick, USA). A constraint of *H* ≥ 1.02 was applied to ensure a zero-slope onset at $t_{min}$. The mean residence time τ was subsequently derived from the fitted model parameters using the first moment of the beta distribution, scaled to the physical time domain:

(5) $$\tau =t_{min}+\frac{H}{H+M}\left(t_{max}-t_{min}\right)$$

This analytical approach provides a robust measure of the mean residence time, insensitive to experimental noise in the signal tails. The corresponding residence time density function E(t) can be derived analytically from the fitted model parameters. For the beta distribution model, the density function is obtained by differentiating Eq. (3) with respect to time, yielding:

(6) $$\begin{array}{c} E\left(t\right)=\frac{1}{\Delta t\cdot B\left(H,M\right)}\cdot {\left(\frac{t-t_{\min}}{\Delta t}\right)}^{H-1}\cdot {\left(\frac{t_{\max}-\;t}{\Delta t}\right)}^{M-1} \end{array}$$

with $\Delta t=t_{max}-t_{min}$.

## Method validation

### Identification of linear range of fluorescence intensity

Fluorescence emission intensity $\left(I_{em}\right)$ depends on five physicochemical and instrumental factors [5]; the intensity of the excitation light $\left(I_{ex}\right)$_,_ the absorption coefficient of the fluorophore (ε), the quantum efficiency (Φ), the analyte concentration (*C*), and the optical path length or sample thickness (*l*):

(7) $$I_{em}\propto \;I_{ex}\cdot \varepsilon \cdot \Phi \cdot C\cdot l$$

This direct proportional relationship is valid only at low concentration. At higher concentrations, $\left(I_{em}\right)$ varies non linearly by physical phenomena such as concentration quenching and the reabsorption of emitted light [20]. To define the linear dynamic range where fluorescence intensity is directly proportional to tracer concentration in the present measurement setup, a systematic characterization of the SF response profile was conducted. A dilution series ranging from 0.023 to 3070 mg·L^-1^ was analyzed.

### Thermal stability of SF

The fluorescence of SF should be independent of thermal degradation of the tracer as this would lead to an unaccountable overlapping effect during RTD assessment, deceiving the measurement at elevated temperature. Table 1 lists the fluorescence intensities of SF measured after the experimental runs with temperature exposure. The listed temperatures represent the maximum in-line temperature reached during processing, while reference samples were collected prior to each run.

**Table 1:** Fluorescence intensities of SF measured after temperature exposure.

Temperature (°C)^a^	Fluorescence intensity (a. u.)^a^	Rel. stability (%)	p-value^b^
Reference (20)	50955 ± 1749	100.0	-
72.4 ± 2.6	50927 ± 1426	100.0	0.92
90.7 ± 2.1	50763 ± 812	99.7	0.67
109.4 ± 2.4	51013 ± 866	100.2	0.90
127.2 ± 2.9	50783 ± 1031	99.7	0.63
144.7 ± 3.0	50483 ± 987	99.1	0.26

a Values are presented as mean ± standard deviation (*N* = 6).

b p-values were calculated using paired *t*-tests comparing the respective temperature treatment to the reference condition (20 °C).

According to Wang'ombe et al [13], SF degrades above 210 °C. They showed in a geothermal setting that even at very high temperature conditions of 325 °C, SF degradation follows first-order kinetics with a decay rate constant of $k_{d}$ ≈ 7.7·10⁻⁶ s⁻¹. This corresponds to a half-life of approximately 25 h, making thermal degradation negligible for RTD measurements in flow-through reactors with residence times in the second domain or below. Our findings are in line with literature, since no statistically significant difference (p > 0.05, *N* = 6 per temperature) was observed in fluorescence intensity before and after heat treatment of the SF tracer solution. This indicates that the tracer remains stable under conditions relevant for high-temperature short-time operations such as HTST or UHT processing.

### Fluorescence intensity at various temperatures and bathochromic shift

**Fig. 3:**
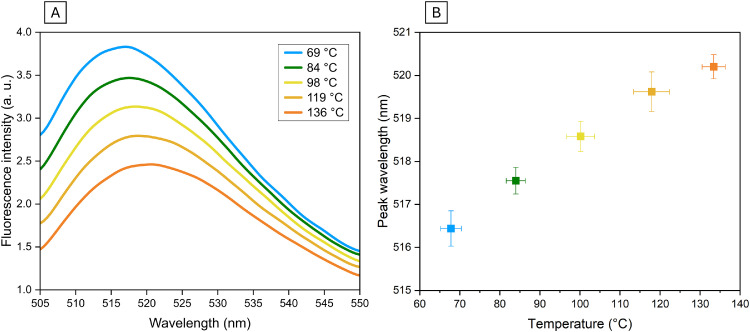
(A) Fluorescence emission spectra of sodium fluorescein recorded at five distinct temperatures between 69 °C and 136 °C. (B) Temperature dependence of the fluorescence peak wavelength of sodium fluorescein. Data points represent the mean values of measurements grouped (*N* = 16 - 24) by experimental temperature setpoints. Error bars denote standard deviations for peak wavelength and temperature.

Fig. 3(A) shows the temperature-dependent fluorescence emission spectra of SF recorded at five distinct temperatures between 69 °C and 136 °C for one run. Additionally, in Fig. 3(B) the bathochromic shift is pictured comprising all data measured. The fluorescence emission spectrum of SF exhibits a moderate decrease in peak intensity with increasing temperature (Fig. 3(A)). The emission curves progressively flattened, resulting in a lower peak maximum at higher temperatures, while the overall spectral shape remains largely preserved. In practical applications where detection hardware may have a limited dynamic range, the moderate signal attenuation of SF ensures that the fluorescence signal remains detectable and quantifiable even at elevated temperatures, avoiding the rapid loss of signal-to-noise ratio that limits the utility of highly temperature-sensitive tracers like Rhodamine B in similar thermal processing environments [17]. However, with increasing temperature, the wavelength spectra flatten progressively, making it difficult to identify the peak reliably and therefore distinguish a precise temperature from single point data. For improved robustness of the method, it is advisable to use the integration of the spectral data instead of absolute peak value for intensity analysis.

Additionally, a peak shift with increasing temperature was observed. The acquired data set was consolidated into five distinct temperature intervals (*N* = 16 - 24 per interval) for display in Fig. 3(B). The peak emission wavelength exhibits a linear correlation with the fluid temperature. Within the investigated range, the peak shifts from approximately 515 nm to 520 nm, corresponding to a bathochromic shift (red shift) with a temperature sensitivity of approximately 0.06 nm·K^-1^ (*N* = 120, R^2^ = 0.92). This value is in good agreement with literature, noting that direct comparisons are subtle. Sjöback et al [15], investigating the absorption spectrum of SF over 4 - 80 °C, reported a shift of 0.07 nm·K⁻¹. Since the emission originates from the same S₁ excited state whose energy is reflected in the absorption peak position, a comparable magnitude of shift in the emission spectrum is physically consistent. Sutton et al [12], who directly measured the emission peak shift of the closely related but structurally distinct dye Fluorescein 27 under 532 nm excitation, report a value of 0.08 nm·K⁻¹ over 24 - 84 °C. Laser-induced fluorescence (LIF) is a widely adopted technique for measuring temperature distributions in liquid systems, exploiting the temperature-dependent fluorescence intensity of dissolved fluorescent dyes. While conventional LIF and DELIF techniques achieve excellent spatial and temporal resolution under moderate thermal conditions, their application to high-temperature continuous flow regimes remain challenging. The experimental setup employed in this study implies a volume-averaged measurement and thus lacking classical microscopic spatial resolution of planar LIF configurations, the results provide critical verification of the dye’s spectral behavior under extended thermal loads. The observed linear bathochromic shift confirms that the fundamental photophysical response of the tracer remains valid even at elevated temperatures and short residence times.

**Fig. 4:**
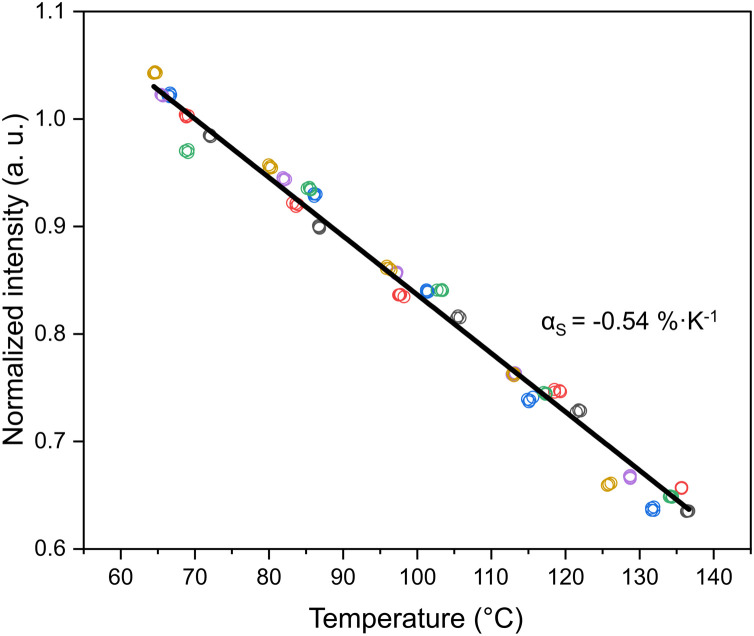
Thermal quenching of the tracer (sodium fluorescein) over a temperature range of 65 °C to 136 °C. Experimental data points (circles, colored by run) are normalized to the intensity at the reference temperature (70 °C). The solid line represents the temperature sensitivity coefficient $\alpha_{S}$.

In Fig. 4, the measured intensity of SF in relation to different temperatures over a range of 65 °C to 136 °C is shown.

For the measured range, the relationship between fluorescence intensity and temperature remained linear, with a decrease of −0.54%·K^-1^ (at 70 °C), denoted as temperature sensitivity coefficient $\alpha_{S}$ (95% CI: −0.554 to −0.535%·K^-1^, *N* = 120, 6 independent runs) and illustrated in Fig. 4. Further published values as reported in literature can be found in Table 2.

**Table 2:** Temperature dependent emission characteristics of SF.

Excitation wavelength (nm)	Temperature dependent emission (%·K^-1^)	Temperature range (°C)	Literature reference
488	−0.3 (at 24 °C)	24 - 46	[27]
514	+2.43 (at 20 °C)	20 - 60	[11]
488	−0.16 (at 20 °C)	20 - 60	[11]
532	+3.22 (at 27 °C)	27 - 80	[28]
490	−0.54 (at 70 °C)	65 - 136	This study

SF exhibits a different temperature sensitivity depending on the excitation wavelength; at $\lambda_{ex}$ >495, absorption increases with temperature, yielding a positive coefficient or weak quenching. At $\lambda_{ex}$ <495, absorption decreases with temperature, yielding a negative coefficient (classical quenching). Measurements taken with different pH buffered aqueous solution show that the temperature dependence at 532 nm is also a function of pH [28]. This provides a possible explanation for the varying emission dependencies that have been reported for the same excitation wavelength of SF. The pH stability (7.4) in this study was maintained across the wide temperature range by choice of the PBS buffer, as outlined earlier.

### Validation of high-temperature RTD characterization and thermal quenching verification

**Fig. 5:**
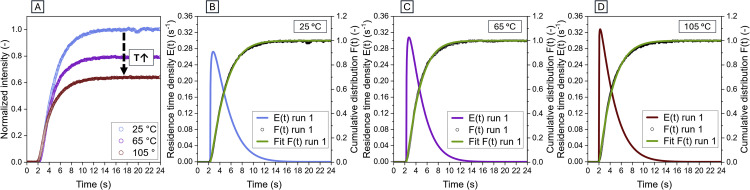
Normalized intensity data from step-input experiments at 25, 65, and 105 °C. (B-D) Experimental cumulative distribution curves F(t) and corresponding fitted semi-empirical model curves (green), and residence time distribution density functions E(t) at the respective temperatures.

To validate the applicability of SF as a tracer for high-temperature residence time characterization, step-response experiments were conducted at 25, 65, and 105 °C (Fig. 5). The semi-empirical beta-distribution model (green solid lines) provided a good fit to the experimental data (black circles) for all tested temperatures (Fig. 5(B-D)). $t_{0}=\;0\;s$ corresponds to the initiation of the step-input switch. The model successfully captured both the initial breakthrough phase and the approach to saturation without systematic deviation. The raw fluorescence intensity profiles as seen in Fig. 5(A) exhibited temperature-dependent quenching, with saturation intensities ($I_{max})$ decreasing significantly as the process temperature increased from 25 °C to 105 °C analogous to the results obtained from spectral data. Despite this reduction in absolute signal-to-noise ratio, the application of the normalization protocol resulted in consistent cumulative distribution curves F(t) across the whole temperature range (Fig. 5 (B-D)). Additionally, Fig. 5(B-D) displays the derived residence time density functions E(t) obtained from the fitted parameters showing similar characteristics for the different temperatures. Table 3 summarizes the hydrodynamic parameters derived from the fitted models. The observed changes in peak height, tailing, and mean residence time are most likely due to temperature-induced variations in fluid properties (e.g., viscosity and density). The shape parameters *M* and *H* remained stable, indicating that the laminar flow regime was preserved. To validate the high-temperature RTD measurements, it was essential to confirm that the observed signal attenuation was exclusively driven by reversible thermal quenching rather than confounding factors such as tracer degradation, photobleaching, or dilution. This was achieved by comparing the steady-state plateau intensities from the RTD experiments with the thermal sensitivity coefficient ($\alpha_{S}$) derived from spectral data. The temperature correction coefficient for the RTD data ($\alpha_{\mathrm{RTD}}$) was determined using the same mathematical methodology as the spectral data coefficient ($\alpha_{S}$). The values obtained showed good agreement, with $\alpha_{RTD}$ = −0.56%·K^-1^ ($\alpha_{S}$ = −0.54%·K^-1^), indicating a consistent thermal dependence across both approaches. A considerable divergence between these values would indicate non-conservative tracer behavior, rendering the standard normalization invalid. The measured temperature dependency makes SF more suitable for tracer studies where there is a significant temperature increase between first and second measurement, compared to, e.g., Rhodamine B (RhB). While RhB exhibits also favorable characteristics for RTD studies in food or pharmaceutical context, an average temperature sensitivity of −1.58%·K^-1^ [12], and −1.2%·K^-1^ [29], respectively, would result in a weak detection signal at high temperatures occurring in UHT processing and other processes at elevated temperatures.

**Table 3:** Fitted hydrodynamic parameters for step-response experiments at different process temperatures.

Set-Temperature (°C)	Replicate	Mean residence time τ (s)	Shape parameter H	Shape parameter M	RMSE
25	1	4.96	1.20	13.9	0.006
	2	4.96	1.20	13.9	0.007
	3	4.95	1.22	14.2	0.006
65	1	4.66	1.21	15.9	0.009
	2	4.67	1.19	15.6	0.010
	3	4.65	1.20	15.6	0.009
105	1	4.44	1.11	14.4	0.010
	2	4.46	1.08	14.0	0.010
	3	4.46	1.07	13.9	0.010

## Limitations

The current findings demonstrate the feasibility of SF-based RTD measurements at elevated temperatures through the validated single-sensor framework. Advancing this concept towards applications in systems with sub-second residence times and pronounced thermal gradients requires further methodological adaptations building on these results. Standard stimulus-response experiments (ideal, instantaneous tracer injection), are inadequate in this regime as the duration of the physically achievable pulse becomes a substantial fraction of the mean residence time, causing the input signal itself to have a distribution that concurs with the system’s true RTD. While this study utilized a single detection cell to validate the tracer method, the established framework lays the foundation for precise characterization of specific reactor modules using a dual-sensor approach. In reactor concepts for high-temperature short-time processing, such as ultra-short pasteurization (USP) or ultra-short sterilization (USS), the heating zone is of special interest [3]. This requires measuring the tracer signal upstream (cold inlet, $I_{in}\left(t\right)$) and downstream (hot outlet, $I_{out}\left(t\right)$) of the unit operation, where $I_{out}\left(t\right)$ is thermally quenched. In this scenario, the characterized temperature sensitivity serves as a critical mass balance verification. By comparing the raw saturation plateaus at the outlet and inlet, and considering the thermal quenching factor, it can be confirmed that the signal reduction is not attributable to otherwise indistinguishable factors such as adsorption or degradation. Furthermore, the necessity for a temperature sensitivity correction becomes an explicit mathematical requirement in circumstances where independent normalization of the detection cell signals is not possible. This may occur, for instance, in pulse-input experiments, or when transient flow conditions prevent the development of a stable saturation plateau at the hot outlet. With temperature effects eliminated, the specific residence time distribution of the reactor can be decoupled from the system’s inlet dispersion using the convolution integral:

(8) $$\begin{array}{c} I_{\mathrm{out},\mathrm{corr}}\left(t\right)=\underset{0}{\int^{t}}I_{\mathrm{in}}\left(t-\tau \right)\cdot E_{\mathrm{reac}\mathrm{tor}}\left(\tau \right)\;d\tau \end{array}$$

The demonstrated stability of the beta-function model suggests that it can serve as a robust parametric transfer function for such deconvolution tasks.

## CRediT author statement

**Dominik Hug**: Conceptualization, Methodology, Software, Validation, Formal analysis, Investigation, Data Curation, Writing - Original Draft, Visualisation, Project administration. **Jonas Meury**: Conceptualization, Methodology, Validation, Investigation, Writing - Review & Editing. **Joseph Dumpler**: Writing - Review & Editing, Supervision. **Corina Sägesser**: Writing - Review & Editing. **Alexander Mathys**: Writing - Review & Editing, Project administration, Funding acquisition, Supervision.

## Data availability

Data underlying the figures in this study are available via the ETH Zurich Research Collection DOI: https://doi.org/10.3929/ethz-c-000799375.

## Declaration of generative AI and AI-assisted technologies in the manuscript preparation process

Generative AI and AI-assisted technologies were used to improve the readability and language of the manuscript. The authors take full responsibility for the content of the publication.

## Declaration of competing interest

The authors declare that they have no known competing financial interests or personal relationships that could have appeared to influence the work reported in this paper.

## References

[1] Adeosun J.T., Lawal A. (2009). Numerical and experimental studies of mixing characteristics in a T-junction microchannel using residence-time distribution. Chem. Eng. Sci..

[2] Deeth H.C., Lewis M.J. (2017).

[3] Mathys A. (2018). Perspective of micro process engineering for thermal food treatment. Front. Nutr..

[4] Cheptea C., Zara A., Ambrosi E., Morosanu A.C., Diaconu M., Miron M., Dorohoi D.O., Dimitriu D.G. (2024). On the solvatochromism of fluorescein sodium. Symmetry (Basel).

[5] Choi Y.J., Sawada K., Narayan R. (2023). Encycl. Sensors Biosens., First Edit.

[6] Weiss T., Slavík M., Bruthans J. (2018). Use of sodium fluorescein dye to visualize the vaporization plane within porous media. J. Hydrol..

[7] Bérard A., Blais B., Patience G.S. (2020). Experimental methods in chemical engineering: residence time distribution—RTD. Can. J. Chem. Eng..

[8] Cruz-Díaz M.R., Rivero E.P., Almazán-Ruiz F.J., Torres-Mendoza Á., González I. (2014). Design of a new FM01-LC reactor in parallel plate configuration using numerical simulation and experimental validation with residence time distribution (RTD). Chem. Eng. Process. Process Intensif..

[9] Mota M.C., Carvalho P., Ramalho J., Leite E. (1991). Spectrophotometric analysis of sodium fluorescein aqueous solutions. Determination of molar absorption coefficient. Int. Ophthalmol..

[10] O’goshi K.I., Serup J. (2006). Safety of sodium fluorescein for in vivo study of skin. Ski. Res. Technol..

[11] Coppeta J., Rogers C. (1998). Dual emission laser induced fluorescence for direct planar scalar behavior measurements. Exp. Fluid..

[12] Sutton J.A., Fisher B.T., Fleming J.W. (2008). A laser-induced fluorescence measurement for aqueous fluid flows with improved temperature sensitivity. Exp. Fluids.

[13] Wang’ombe B., Okiambe E., Omenda P., Rathore I.V.S., Ambusso W. (2014). A numerical solution to estimate hydro-geologic parameters of a fractured geothermal porous medium based on fluorescein thermal decay correction. Geothermics.

[14] Guan X., Su Z. (2008). Synthesis and characterization of fluorescent starch using fluorescein as fluorophore: potential polymeric temperature/pH indicators. Polym. Adv. Technol..

[15] Sjöback R., Nygren J., Kubista M. (1995). Absorption and fluorescence properties of fluorescein. Spectrochim. Acta Part A Mol. Spectrosc..

[16] Eghtesad A., Bijarchi M.A., Shafii M.B., Afshin H. (2024). A state-of-the-art review on laser-induced fluorescence (LIF) method with application in temperature measurement. Int. J. Therm. Sci..

[17] Toriyama K., Funatani S., Tada S. (2024). Development of a DualEmission laser-induced fluorescence (DELIF) method for long-term temperature measurements. Sensors.

[18] De Bonis A., Galasso A., Ibris N., Sansone M., Santagata A., Teghil R. (2012). Ultra-short pulsed laser deposition of thin silver films for surface enhanced raman scattering. Surf. Coat. Technol..

[19] Ham J.H., Platzer B. (2004). Semi-empirical equations for residence time distributions in disperse systems - part 1: continuous phase. Chem. Eng. Technol..

[20] Liu M., Jia M., Pan H., Li L., Chang M., Ren H., Argoul F., Zhang S., Xu J. (2014). Instrument response standard in time-resolved fluorescence spectroscopy at visible wavelength: quenched fluorescein sodium. Appl. Spectrosc..

[21] Doughty M.J. (2010). PH dependent spectral properties of sodium fluorescein ophthalmic solutions revisited. Ophthalm. Physiol. Opt..

[22] Käss W. (2018). Tracing technique in geohydrology.

[23] Bandura A.V., Lvov S.N. (2006). The ionization constant of water over wide ranges of temperature and density. J. Phys. Chem. Ref. Data.

[24] Marshall W.L., Franck E.U. (1981). Ion product of water substance, 0–1000°C, 1–10,000 bars new international formulation and its background. J. Phys. Chem. Ref. Data.

[25] Reineke K., Mathys A., Knorr D. (2011). Shift of pH-value during thermal treatments in buffer solutions and selected foods. Int. J. Food Prop..

[26] Lakowicz J.R. (2006).

[27] Walker D.A. (1987). A fluorescence technique for measurement of concentration in mixing liquids. J. Phys. E. Sci. Instrum..

[28] Chaze W., Caballina O., Castanet G., Lemoine F. (2016). The saturation of the fluorescence and its consequences for laser-induced fluorescence thermometry in liquid flows. Exp. Fluid..

[29] Kan T., Aoki H., Binh-Khiem N., Matsumoto K., Shimoyama I. (2013). Ratiometric optical temperature sensor using two fluorescent dyes dissolved in an ionic liquid encapsulated by parylene film. Sensors.

